# The Giant Resin Bee, *Megachile sculpturalis* Smith: New Distributional Records for the Mid- and Gulf-south USA

**DOI:** 10.3897/BDJ.3.e6733

**Published:** 2015-10-26

**Authors:** Katherine A. Parys, Amber D. Tripodi, Blair J. Sampson

**Affiliations:** ‡ USDA-ARS Southern Insect Management Research Unit, Stoneville, MS, United States of America; § USDA-ARS Pollinating Insect: Biology, Management and Systematics Research Unit, Logan, UT, United States of America; | USDA-ARS Southern Horticultural Research Unit, Poplarville, MS, United States of America

**Keywords:** Callomegachile, adventive, introduced, invasive species, distribution, range expansion, Giant Resin Bee, *Megachile sculpturalis*

## Abstract

**Background:**

Megachile (Callomegachile) sculpturalis Smith, the giant resin bee, is an adventive species in the United States. First established in the United States during the early 1990s, records currently exist from most states east of the Mississippi River along with Iowa and Kansas.

**New information:**

New distributional records are presented for Megachile (Callomegachile) sculpturalis Smith, an introduced bee. Additional records presented here expand the known distribution southwest through Arkansas, Louisiana, Mississippi, Missouri, and Texas. An updated host plant list containing new records is also presented, expanding the number of known floral associations.

## Introduction

The giant resin bee, Megachile (Callomegachile) sculpturalis Smith, is a large, distinctive bee, adventive to the United States and Europe (Hinojosa-Díaz 2008, Quaranta et al. 2014). Originally found throughout the eastern Palearctic and Oriental regions including Japan, China, and other parts of eastern Asia, it was introduced into the United States and first collected from North Carolina in 1994 (Mangum and Brooks 1997). The range rapidly expanded across North America from the original location, reaching southwest to Alabama by 1999, north to Canada by 2002, northwest to Wisconsin by 2004, northeast to Maine by 2008, and westward to Kansas by 2008 (Hinojosa-Díaz 2008, Kondo et al. 2000, Mangum and Brooks 1997, Mangum and Sumner 2003, Mazurkiewicz 2010, Paiero and Buck 2003, Wolf and Ascher 2008).

The previously known distribution included most states east of the Mississippi River, with the exception of Mississippi (Mazurkiewicz 2010, Maier 2005, Tonietto and Ascher 2008). Niche modeling predicted that the western edge of range expansion will eventually stretch to western Texas, Oklahoma, Kansas, Nebraska, and South Dakota, areas of the western coast, along with sections of Mexico and the West Indies (Hinojosa-Díaz et al. 2005). This species has also been recently introduced into Europe and is currently established in France, Switzerland, and Italy (Quaranta et al. 2014, Amiet 2012, Vereecken and Barbier 2009, Gihr and Westrich 2013). *Megachile sculpturalis* is polylectic, but it has been speculated that they may preferentially pollinate plants that have been introduced into North America from its native range (Laport and Minckley 2012). Females prefer to nest in sites located in cavities (i.e. hollow stems or holes made by other insects) in shaded areas, and at least 0.5 m above the ground (Iwata 1933).

Characters to differentiate *M. sculpturalis* from native megachilids are presented in (Michener 2007), but it is currently the only species in the subgenus *Callomegachile* Michener widely established in the Nearctic region. Easily differentiated from native bees by a large, narrow, elongated body, female *M. sculpturalis* range in size from 22 – 27 mm, while males are considerably smaller at 14 – 19 mm with a distinctly wide yellowish “moustache” on their lower face; both sexes have prominently infuscated wings (Fig. 1) (Batra 1998). An additional species in the same subgenus as *M. sculpturalis*, *Megachile umbripennis* Smith, has been recently observed in southern Florida and New Jersey, but this bee is considerably smaller in size than *M. sculpturalis* (Ascher and Pickering 2015. Another relative of *M. sculpturalis*, Megachile (Callomegachile) rufipennis F., originally from Africa has become established in the Greater Antilles, but has not been observed in Nearctic areas (Genaro 1996, Pasteels 1965).

There are currently 19 species of introduced megachilid bees in the continental United States, including *M. sculpturalis* (Droge 2015). As introductions of non-native species have continued, evidence that some species pose threats to biodiversity and native ecosystems has increased (Simberloff et al. 2013). Noting the distribution, presence, and establishment of adventive species is important for documenting future impacts on native communities (Cane 2003). *Megachile sculpturalis* is a xylophilous (wood-loving) bee; however, females are incapable of boring their own cavities in wood. Instead, they are known to occupy abandoned nests of similarly sized bees, notably the nests of native carpenter bees, *Xylocopa virginica* (L.) (Batra 1998, Mangum and Brooks 1997). More recently, female *M. sculpturalis* have been observed aggressively evicting carpenter bee females from their nests (Laport and Minckley 2012, Roulston and Malfi 2012). Within their native range *M. sculpturalis* occupy nesting sites abandoned by a variety of other species (Iwata 1933).

## Materials and methods

Collection data from adult specimens *of M. sculpturalis* taken in the mid- and gulf-south, in addition to specimens from Florida and Michigan, were gathered from both institutions and personal collections. Institutional collections used in the manuscript are listed below; acronyms, when available, follow Evenhuis (2015) and the global registry of biodiversity repositories (GRBio 2015). Personal collections from research studies include those of Mike Arduser (MA; surveys from Missouri), Zach Scott (ZS; survey data from Rhode Island), and the authors (designated by initials), and are designated as such.

**UAAM** - The Arthropod Museum, University of Arkansas, Department of Entomology, Fayetteville, AR

**MEM** - Mississippi Entomological Museum, Mississippi State University, Department of Biochemistry, Molecular Biology, Entomology, & Plant Pathology, Mississippi State, MS

**SFAC** - Stephen F. Austin State University, Department of Biology, Nacogdoches, TX

**UMIC** - University of Mississippi, Department of Biology, University, MS

**BBSL** - United States Department of Agriculture-Agricultural Research Service, Bee Biology and Systematics Laboratory, Logan, UT

## Taxon treatments

### Megachile (Callomegachile) sculpturalis

Smith, 1853

#### Materials

- **Type status:** Other material. **Occurrence:** recordedBy: Amber D. Tripodi; individualCount: 1; sex: male; lifeStage: adult; occurrenceStatus: present; preparations: whole animal (pinned); disposition: in collection; **Taxon:** scientificName: *Megachile sculpturalis* Smith, 1853; kingdom: Animalia; phylum: Arthropoda; class: Insecta; order: Hymenoptera; family: Megachilidae; genus: Megachile; subgenus: Callomegachile; specificEpithet: sculpturalis; scientificNameAuthorship: Smith, 1853; vernacularName: Giant Resin Bee; nomenclaturalCode: ICZN; taxonomicStatus: accepted; **Location:** continent: North America; country: United States; countryCode: US; stateProvince: Arkansas; county: Benton; municipality: Gentry; locality: Chesney Prairie; decimalLatitude: 36.221636; decimalLongitude: -94.484357; **Identification:** identifiedBy: Amber D. Tripodi; **Event:** year: 2012; month: 6; day: 20; verbatimEventDate: 20-6-2012; **Record Level:** type: PhysicalObject; language: en; institutionCode: BBSL; basisOfRecord: PreservedSpecimen
- **Type status:** Other material. **Occurrence:** recordedBy: Amber D. Tripodi; individualCount: 1; sex: male; lifeStage: adult; occurrenceStatus: present; preparations: whole animal (pinned); disposition: in collection; **Taxon:** scientificName: *Megachile sculpturalis* Smith, 1853; kingdom: Animalia; phylum: Arthropoda; class: Insecta; order: Hymenoptera; family: Megachilidae; genus: Megachile; subgenus: Callomegachile; specificEpithet: sculpturalis; scientificNameAuthorship: Smith, 1853; vernacularName: Giant Resin Bee; nomenclaturalCode: ICZN; taxonomicStatus: accepted; **Location:** continent: North America; country: United States; countryCode: US; stateProvince: Arkansas; county: Benton; municipality: Rogers; locality: Searles Prairie; decimalLatitude: 36.356395; decimalLongitude: -94.144186; **Identification:** identifiedBy: Amber D. Tripodi; **Event:** year: 2012; month: 6; day: 20; verbatimEventDate: 20-6-2012; **Record Level:** type: PhysicalObject; language: en; institutionCode: BBSL; basisOfRecord: PreservedSpecimen
- **Type status:** Other material. **Occurrence:** recordedBy: Amber D. Tripodi; individualCount: 1; sex: female; lifeStage: adult; occurrenceStatus: present; preparations: whole animal (pinned); disposition: in collection; **Taxon:** scientificName: *Megachile sculpturalis* Smith, 1853; kingdom: Animalia; phylum: Arthropoda; class: Insecta; order: Hymenoptera; family: Megachilidae; genus: Megachile; subgenus: Callomegachile; specificEpithet: sculpturalis; scientificNameAuthorship: Smith, 1853; vernacularName: Giant Resin Bee; nomenclaturalCode: ICZN; taxonomicStatus: accepted; **Location:** continent: North America; country: United States; countryCode: US; stateProvince: Arkansas; county: Madison; municipality: Hindsville; decimalLatitude: 36.206389; decimalLongitude: -93.850278; **Identification:** identifiedBy: Amber D. Tripodi; **Event:** samplingProtocol: Xylocopa virginica trap; year: 2012; month: 6; day: 19; verbatimEventDate: 19-6-2012; **Record Level:** type: PhysicalObject; language: en; institutionCode: BBSL; basisOfRecord: PreservedSpecimen
- **Type status:** Other material. **Occurrence:** recordedBy: T. D. Edwards; individualCount: 1; sex: female; lifeStage: adult; occurrenceStatus: present; preparations: whole animal (pinned); disposition: in collection; **Taxon:** scientificName: *Megachile sculpturalis* Smith, 1853; kingdom: Animalia; phylum: Arthropoda; class: Insecta; order: Hymenoptera; family: Megachilidae; genus: Megachile; subgenus: Callomegachile; specificEpithet: sculpturalis; scientificNameAuthorship: Smith, 1853; vernacularName: Giant Resin Bee; nomenclaturalCode: ICZN; taxonomicStatus: accepted; **Location:** continent: North America; country: United States; countryCode: US; stateProvince: Arkansas; county: Washinton; municipality: Fayetteville; locality: University of Arkansas; verbatimCoordinates: 36° 04’ 20.24 N, 94° 10’ 24.98 W; verbatimLatitude: 36° 04’ 20.24 N; verbatimLongitude: 94° 10’ 24.98 W; decimalLatitude: 36.072289; decimalLongitude: -94.173606; **Event:** year: 2012; month: 6; day: 2; verbatimEventDate: 2.VI.2012; **Record Level:** type: PhysicalObject; language: en; institutionCode: UAAM; basisOfRecord: PreservedSpecimen
- **Type status:** Other material. **Occurrence:** recordedBy: Amber D. Tripodi; individualCount: 1; sex: female; lifeStage: adult; occurrenceStatus: present; preparations: whole animal (pinned); disposition: in collection; **Taxon:** scientificName: *Megachile sculpturalis* Smith, 1853; kingdom: Animalia; phylum: Arthropoda; class: Insecta; order: Hymenoptera; family: Megachilidae; genus: Megachile; subgenus: Callomegachile; specificEpithet: sculpturalis; scientificNameAuthorship: Smith, 1853; vernacularName: Giant Resin Bee; nomenclaturalCode: ICZN; taxonomicStatus: accepted; **Location:** continent: North America; country: United States; countryCode: US; stateProvince: Arkansas; county: Washinton; municipality: Fayetteville; locality: World Peace Wetland Prairie; decimalLatitude: 36.051894; decimalLongitude: -94.172728; **Identification:** identifiedBy: Amber D. Tripodi; **Event:** year: 2011; month: 6; day: 29; verbatimEventDate: Jun-29-2011; **Record Level:** type: PhysicalObject; institutionCode: UAAM; basisOfRecord: PreservedSpecimen
- **Type status:** Other material. **Occurrence:** recordedBy: Clinton E. Trammel & Amber D. Tripodi; individualCount: 1; sex: female; lifeStage: adult; occurrenceStatus: present; preparations: whole animal (pinned); disposition: in collection; **Taxon:** scientificName: *Megachile sculpturalis* Smith, 1853; kingdom: Animalia; phylum: Arthropoda; class: Insecta; order: Hymenoptera; family: Megachilidae; genus: Megachile; subgenus: Callomegachile; specificEpithet: sculpturalis; scientificNameAuthorship: Smith, 1853; vernacularName: Giant Resin Bee; nomenclaturalCode: ICZN; taxonomicStatus: accepted; **Location:** continent: North America; country: United States; countryCode: US; stateProvince: Florida; county: Alachua; municipality: Gainesville; locality: University of Florida; verbatimCoordinates: 29.650428, -82.342365; verbatimLatitude: 29.650428; verbatimLongitude: -82.342365; decimalLatitude: 29.650428; decimalLongitude: -82.342365; **Identification:** identifiedBy: Amber D. Tripodi; **Event:** year: 2013; month: 7; day: 8; verbatimEventDate: Jul-08-2013; **Record Level:** type: PhysicalObject; institutionCode: BBSL; basisOfRecord: PreservedSpecimen
- **Type status:** Other material. **Occurrence:** recordedBy: Clinton E. Trammel & Amber D. Tripodi; individualCount: 12; sex: female; lifeStage: adult; occurrenceStatus: present; preparations: whole animal (pinned); disposition: in collection; **Taxon:** scientificName: *Megachile sculpturalis* Smith, 1853; kingdom: Animalia; phylum: Arthropoda; class: Insecta; order: Hymenoptera; family: Megachilidae; genus: Megachile; subgenus: Callomegachile; specificEpithet: sculpturalis; scientificNameAuthorship: Smith, 1853; vernacularName: Giant Resin Bee; nomenclaturalCode: ICZN; taxonomicStatus: accepted; **Location:** continent: North America; country: United States; countryCode: US; stateProvince: Florida; county: Alachua; municipality: Gainesville; locality: University of Florida; verbatimCoordinates: 29.650428, -82.342365; verbatimLatitude: 29.650428; verbatimLongitude: -82.342365; decimalLatitude: 29.650428; decimalLongitude: -82.342365; **Identification:** identifiedBy: Amber D. Tripodi; **Event:** year: 2013; month: 7; day: 8; verbatimEventDate: Jul-08-2013; **Record Level:** type: PhysicalObject; institutionCode: ADT; basisOfRecord: PreservedSpecimen
- **Type status:** Other material. **Occurrence:** recordedBy: Amber D. Tripodi; individualCount: 3; sex: 1 male, 2 female; lifeStage: adult; occurrenceStatus: present; preparations: whole animal (pinned); disposition: in collection; **Taxon:** scientificName: *Megachile sculpturalis* Smith, 1853; kingdom: Animalia; phylum: Arthropoda; class: Insecta; order: Hymenoptera; family: Megachilidae; genus: Megachile; subgenus: Callomegachile; specificEpithet: sculpturalis; scientificNameAuthorship: Smith, 1853; vernacularName: Giant Resin Bee; nomenclaturalCode: ICZN; taxonomicStatus: accepted; **Location:** continent: North America; country: United States; countryCode: US; stateProvince: Florida; county: Alachua; municipality: Gainesville; locality: University of Florida; verbatimCoordinates: 29.650428, -82.342365; verbatimLatitude: 29.650428; verbatimLongitude: -82.342365; decimalLatitude: 29.650428; decimalLongitude: -82.342365; **Identification:** identifiedBy: Amber D. Tripodi; **Event:** year: 2013; month: 6; day: 21; verbatimEventDate: Jun-21-2015; **Record Level:** type: PhysicalObject; institutionCode: BBSL; basisOfRecord: PreservedSpecimen
- **Type status:** Other material. **Occurrence:** recordedBy: Amber D. Tripodi; individualCount: 59; sex: 2 male, 57 female; lifeStage: adult; occurrenceStatus: present; preparations: whole animal (pinned); disposition: in collection; **Taxon:** scientificName: *Megachile sculpturalis* Smith, 1853; kingdom: Animalia; phylum: Arthropoda; class: Insecta; order: Hymenoptera; family: Megachilidae; genus: Megachile; subgenus: Callomegachile; specificEpithet: sculpturalis; scientificNameAuthorship: Smith, 1853; vernacularName: Giant Resin Bee; nomenclaturalCode: ICZN; taxonomicStatus: accepted; **Location:** continent: North America; country: United States; countryCode: US; stateProvince: Florida; county: Alachua; municipality: Gainesville; locality: University of Florida; verbatimCoordinates: 29.650428, -82.342365; verbatimLatitude: 29.650428; verbatimLongitude: -82.342365; decimalLatitude: 29.650428; decimalLongitude: -82.342365; **Identification:** identifiedBy: Amber D. Tripodi; **Event:** year: 2013; month: 6; day: 21; verbatimEventDate: Jun-21-2015; **Record Level:** type: PhysicalObject; institutionCode: ADT; basisOfRecord: PreservedSpecimen
- **Type status:** Other material. **Occurrence:** recordedBy: Amber D. Tripodi; individualCount: 1; sex: female; lifeStage: adult; occurrenceStatus: present; preparations: whole animal (pinned); disposition: in collection; **Taxon:** scientificName: *Megachile sculpturalis* Smith, 1853; kingdom: Animalia; phylum: Arthropoda; class: Insecta; order: Hymenoptera; family: Megachilidae; genus: Megachile; subgenus: Callomegachile; specificEpithet: sculpturalis; scientificNameAuthorship: Smith, 1853; vernacularName: Giant Resin Bee; nomenclaturalCode: ICZN; taxonomicStatus: accepted; **Location:** continent: North America; country: United States; countryCode: US; stateProvince: Louisiana; county: West Feliciana; municipality: St. Francisville; decimalLatitude: 30.792733; decimalLongitude: -91.249833; **Identification:** identifiedBy: Amber D. Tripodi; **Event:** year: 2013; month: 7; day: 4; verbatimEventDate: Jul-04-2013; **Record Level:** type: PhysicalObject; institutionCode: ADT; basisOfRecord: PreservedSpecimen
- **Type status:** Other material. **Occurrence:** recordedBy: Amber D. Tripodi; individualCount: 1; sex: female; lifeStage: adult; occurrenceStatus: present; preparations: whole animal (pinned); disposition: in collection; **Taxon:** scientificName: *Megachile sculpturalis* Smith, 1853; kingdom: Animalia; phylum: Arthropoda; class: Insecta; order: Hymenoptera; family: Megachilidae; genus: Megachile; subgenus: Callomegachile; specificEpithet: sculpturalis; scientificNameAuthorship: Smith, 1853; vernacularName: Giant Resin Bee; nomenclaturalCode: ICZN; taxonomicStatus: accepted; **Location:** continent: North America; country: United States; countryCode: US; stateProvince: Michigan; county: Berrien; municipality: Watervlivet; verbatimCoordinates: 42.179430, -86.258730; verbatimLatitude: 42.179430; verbatimLongitude: -86.258730; decimalLatitude: 42.179430; decimalLongitude: -86.258730; **Identification:** identifiedBy: Amber D. Tripodi; **Event:** year: 2015; month: 7; day: 31; verbatimEventDate: Jul-31-2015; **Record Level:** type: PhysicalObject; institutionCode: BBSL; basisOfRecord: PreservedSpecimen
- **Type status:** Other material. **Occurrence:** recordedBy: Amber D. Tripodi; individualCount: 11; sex: female; lifeStage: adult; occurrenceStatus: present; preparations: whole animal (pinned); disposition: in collection; **Taxon:** scientificName: *Megachile sculpturalis* Smith, 1853; kingdom: Animalia; phylum: Arthropoda; class: Insecta; order: Hymenoptera; family: Megachilidae; genus: Megachile; subgenus: Callomegachile; specificEpithet: sculpturalis; scientificNameAuthorship: Smith, 1853; vernacularName: Giant Resin Bee; nomenclaturalCode: ICZN; taxonomicStatus: accepted; **Location:** continent: North America; country: United States; countryCode: US; stateProvince: Michigan; county: Berrien; municipality: Watervlivet; verbatimCoordinates: 42.179430, -86.258730; verbatimLatitude: 42.179430; verbatimLongitude: -86.258730; decimalLatitude: 42.179430; decimalLongitude: -86.258730; **Identification:** identifiedBy: Amber D. Tripodi; **Event:** year: 2015; month: 7; day: 31; verbatimEventDate: Jul-31-2015; **Record Level:** type: PhysicalObject; institutionCode: ADT; basisOfRecord: PreservedSpecimen
- **Type status:** Other material. **Occurrence:** recordedBy: M. L. Whitten; individualCount: 3; sex: female; lifeStage: adult; occurrenceStatus: present; preparations: whole animal (pinned); disposition: in collection; **Taxon:** scientificName: *Megachile sculpturalis* Smith, 1853; kingdom: Animalia; phylum: Arthropoda; class: Insecta; order: Hymenoptera; family: Megachilidae; genus: Megachile; subgenus: Callomegachile; specificEpithet: sculpturalis; scientificNameAuthorship: Smith, 1853; vernacularName: Giant Resin Bee; nomenclaturalCode: ICZN; taxonomicStatus: accepted; **Location:** continent: North America; country: United States; countryCode: US; stateProvince: Mississippi; county: Grenada; municipality: Scobey; locality: Cascilla Road; locationRemarks: old wooden goat barn, nesting in holes made by Xylocopa; verbatimCoordinates: 33°53’34” N, -89°55’09” W; verbatimLatitude: 33°53’34” N; verbatimLongitude: -89°55’09” W; decimalLatitude: 33.892778; decimalLongitude: -89.919167; **Identification:** identifiedBy: Katherine A. Parys; **Event:** samplingProtocol: at large; year: 2015; month: 7; day: 4; verbatimEventDate: Jul-04-2015; **Record Level:** type: PhysicalObject; institutionCode: BBSL; basisOfRecord: PreservedSpecimen
- **Type status:** Other material. **Occurrence:** recordedBy: M. L. Whitten; individualCount: 3; sex: female; lifeStage: adult; occurrenceStatus: present; preparations: whole animal (pinned); disposition: in collection; **Taxon:** scientificName: *Megachile sculpturalis* Smith, 1853; kingdom: Animalia; phylum: Arthropoda; class: Insecta; order: Hymenoptera; family: Megachilidae; genus: Megachile; subgenus: Callomegachile; specificEpithet: sculpturalis; scientificNameAuthorship: Smith, 1853; vernacularName: Giant Resin Bee; nomenclaturalCode: ICZN; taxonomicStatus: accepted; **Location:** continent: North America; country: United States; countryCode: US; stateProvince: Mississippi; county: Grenada; municipality: Scobey; locality: Cascilla Road; locationRemarks: old wooden goat barn, nesting in holes made by Xylocopa; verbatimCoordinates: 33°53’34” N, -89°55’09” W; verbatimLatitude: 33°53’34” N; verbatimLongitude: -89°55’09” W; decimalLatitude: 33.892778; decimalLongitude: -89.919167; **Identification:** identifiedBy: Katherine A. Parys; **Event:** samplingProtocol: at large; year: 2015; month: 7; day: 4; verbatimEventDate: Jul-04-2015; **Record Level:** type: PhysicalObject; institutionCode: MEM; basisOfRecord: PreservedSpecimen
- **Type status:** Other material. **Occurrence:** recordedBy: M. L. Whitten; individualCount: 2; sex: female; lifeStage: adult; occurrenceStatus: present; preparations: whole animal (pinned); disposition: in collection; **Taxon:** scientificName: *Megachile sculpturalis* Smith, 1853; kingdom: Animalia; phylum: Arthropoda; class: Insecta; order: Hymenoptera; family: Megachilidae; genus: Megachile; subgenus: Callomegachile; specificEpithet: sculpturalis; scientificNameAuthorship: Smith, 1853; vernacularName: Giant Resin Bee; nomenclaturalCode: ICZN; taxonomicStatus: accepted; **Location:** continent: North America; country: United States; countryCode: US; stateProvince: Mississippi; county: Grenada; municipality: Scobey; locality: Cascilla Road; locationRemarks: old wooden goat barn, nesting in holes made by Xylocopa; verbatimCoordinates: 33°53’34” N, -89°55’09” W; verbatimLatitude: 33°53’34” N; verbatimLongitude: -89°55’09” W; decimalLatitude: 33.892778; decimalLongitude: -89.919167; **Identification:** identifiedBy: Katherine A. Parys; **Event:** samplingProtocol: at large; year: 2015; month: 7; day: 4; verbatimEventDate: Jul-04-2015; **Record Level:** type: PhysicalObject; institutionCode: KAP; basisOfRecord: PreservedSpecimen
- **Type status:** Other material. **Occurrence:** individualCount: 1; sex: female; lifeStage: adult; occurrenceStatus: present; preparations: whole animal (pinned); disposition: in collection; **Taxon:** scientificName: *Megachile sculpturalis* Smith, 1853; kingdom: Animalia; phylum: Arthropoda; class: Insecta; order: Hymenoptera; family: Megachilidae; genus: Megachile; subgenus: Callomegachile; specificEpithet: sculpturalis; scientificNameAuthorship: Smith, 1853; vernacularName: Giant Resin Bee; nomenclaturalCode: ICZN; taxonomicStatus: accepted; **Location:** continent: North America; country: United States; countryCode: US; stateProvince: Mississippi; county: Lafayette; municipality: Oxford; **Event:** year: 2005; month: 7; day: 25; verbatimEventDate: Jul-25-2005; **Record Level:** type: PhysicalObject; institutionCode: UMIC; basisOfRecord: PreservedSpecimen
- **Type status:** Other material. **Occurrence:** recordedBy: Jonas King; individualCount: 7; sex: male; lifeStage: adult; occurrenceStatus: present; preparations: whole animal (pinned); disposition: in collection; **Taxon:** scientificName: *Megachile sculpturalis* Smith, 1853; kingdom: Animalia; phylum: Arthropoda; class: Insecta; order: Hymenoptera; family: Megachilidae; genus: Megachile; subgenus: Callomegachile; specificEpithet: sculpturalis; scientificNameAuthorship: Smith, 1853; vernacularName: Giant Resin Bee; nomenclaturalCode: ICZN; taxonomicStatus: accepted; **Location:** continent: North America; country: United States; countryCode: US; stateProvince: Mississippi; county: Lafayette; municipality: Oxford; locality: University of Mississippi Campus; locationRemarks: taken from golden raintree, Koelreuteria paniculata; **Event:** year: 2008; month: 4; day: 15; verbatimEventDate: Apr-15-2008; **Record Level:** type: PhysicalObject; institutionCode: UMIC; basisOfRecord: PreservedSpecimen
- **Type status:** Other material. **Occurrence:** recordedBy: Clinton E. Trammel & Amber D. Tripodi; individualCount: 2; sex: 1 male, 1 female; lifeStage: adult; occurrenceStatus: present; preparations: whole animal (pinned); disposition: in collection; **Taxon:** scientificName: *Megachile sculpturalis* Smith, 1853; kingdom: Animalia; phylum: Arthropoda; class: Insecta; order: Hymenoptera; family: Megachilidae; genus: Megachile; subgenus: Callomegachile; specificEpithet: sculpturalis; scientificNameAuthorship: Smith, 1853; vernacularName: Giant Resin Bee; nomenclaturalCode: ICZN; taxonomicStatus: accepted; **Location:** continent: North America; country: United States; countryCode: US; stateProvince: Mississippi; county: Lafayette; municipality: Oxford; locality: University of Mississippi Campus; **Identification:** identifiedBy: Amber D. Tripodi; **Event:** year: 2013; month: 7; day: 12; verbatimEventDate: Jul-12-2013; **Record Level:** type: PhysicalObject; institutionCode: BBSL; basisOfRecord: PreservedSpecimen
- **Type status:** Other material. **Occurrence:** recordedBy: Clinton E. Trammel & Amber D. Tripodi; individualCount: 4; sex: 2 male, 2 female; lifeStage: adult; occurrenceStatus: present; preparations: whole animal (pinned); disposition: in collection; **Taxon:** scientificName: *Megachile sculpturalis* Smith, 1853; kingdom: Animalia; phylum: Arthropoda; class: Insecta; order: Hymenoptera; family: Megachilidae; genus: Megachile; subgenus: Callomegachile; specificEpithet: sculpturalis; scientificNameAuthorship: Smith, 1853; vernacularName: Giant Resin Bee; nomenclaturalCode: ICZN; taxonomicStatus: accepted; **Location:** continent: North America; country: United States; countryCode: US; stateProvince: Mississippi; county: Lafayette; municipality: Oxford; locality: University of Mississippi Campus; decimalLatitude: 34.365225; decimalLongitude: -89.534050; **Identification:** identifiedBy: Amber D. Tripodi; **Event:** year: 2013; month: 7; day: 12; verbatimEventDate: Jul-12-2013; **Record Level:** type: PhysicalObject; institutionCode: ADT; basisOfRecord: PreservedSpecimen
- **Type status:** Other material. **Occurrence:** recordedBy: John Austin Coleman and Severino Signa; individualCount: 1; sex: female; lifeStage: adult; occurrenceStatus: present; preparations: whole animal (pinned); disposition: in collection; **Taxon:** scientificName: *Megachile sculpturalis* Smith, 1853; kingdom: Animalia; phylum: Arthropoda; class: Insecta; order: Hymenoptera; family: Megachilidae; genus: Megachile; subgenus: Callomegachile; specificEpithet: sculpturalis; scientificNameAuthorship: Smith, 1853; vernacularName: Giant Resin Bee; nomenclaturalCode: ICZN; taxonomicStatus: accepted; **Location:** continent: North America; country: United States; countryCode: US; stateProvince: Mississippi; county: Tallahatchie; municipality: Paynes; locality: Shook Rd and Hwy 35; **Identification:** identifiedBy: Katherine A. Parys; **Event:** samplingProtocol: Bycatch in pheromone trap for moths; year: 2015; month: 5; day: 23; verbatimEventDate: May-23-2015; **Record Level:** type: PhysicalObject; institutionCode: MEM; basisOfRecord: PreservedSpecimen
- **Type status:** Other material. **Occurrence:** recordedBy: Katherine A. Parys and Nathan S. Little; individualCount: 2; sex: female; lifeStage: adult; occurrenceStatus: present; preparations: whole animal (pinned); disposition: in collection; **Taxon:** scientificName: *Megachile sculpturalis* Smith, 1853; kingdom: Animalia; phylum: Arthropoda; class: Insecta; order: Hymenoptera; family: Megachilidae; genus: Megachile; subgenus: Callomegachile; specificEpithet: sculpturalis; scientificNameAuthorship: Smith, 1853; vernacularName: Giant Resin Bee; nomenclaturalCode: ICZN; taxonomicStatus: accepted; **Location:** continent: North America; country: United States; countryCode: US; stateProvince: Mississippi; county: Tallahatchie; municipality: Paynes; locationRemarks: old pig barn, nesting in holes made by Xylocopa; verbatimCoordinates: 33°55’29” N, -90°03’53” W; verbatimLatitude: 33°55’29” N; verbatimLongitude: -90°03’53” W; decimalLatitude: 33.924722; decimalLongitude: -90.064722; **Identification:** identifiedBy: Katherine A. Parys; **Event:** samplingProtocol: by net; year: 2015; month: 7; day: 6; verbatimEventDate: Jul-06-2015; **Record Level:** type: PhysicalObject; institutionCode: BBSL; basisOfRecord: PreservedSpecimen
- **Type status:** Other material. **Occurrence:** recordedBy: Katherine A. Parys and Nathan S. Little; individualCount: 2; sex: female; lifeStage: adult; occurrenceStatus: present; preparations: whole animal (pinned); disposition: in collection; **Taxon:** scientificName: *Megachile sculpturalis* Smith, 1853; kingdom: Animalia; phylum: Arthropoda; class: Insecta; order: Hymenoptera; family: Megachilidae; genus: Megachile; subgenus: Callomegachile; specificEpithet: sculpturalis; scientificNameAuthorship: Smith, 1853; vernacularName: Giant Resin Bee; nomenclaturalCode: ICZN; taxonomicStatus: accepted; **Location:** continent: North America; country: United States; countryCode: US; stateProvince: Mississippi; county: Tallahatchie; municipality: Paynes; locationRemarks: old pig barn, nesting in holes made by Xylocopa; verbatimCoordinates: 33°55’29” N, -90°03’53” W; verbatimLatitude: 33°55’29” N; verbatimLongitude: -90°03’53” W; decimalLatitude: 33.924722; decimalLongitude: -90.064722; **Identification:** identifiedBy: Katherine A. Parys; **Event:** samplingProtocol: by net; year: 2015; month: 7; day: 6; verbatimEventDate: Jul-06-2015; **Record Level:** type: PhysicalObject; institutionCode: MEM; basisOfRecord: PreservedSpecimen
- **Type status:** Other material. **Occurrence:** recordedBy: JoVonn G. Hill; individualCount: 2; sex: female; lifeStage: adult; occurrenceStatus: present; preparations: whole animal (pinned); disposition: in collection; **Taxon:** scientificName: *Megachile sculpturalis* Smith, 1853; kingdom: Animalia; phylum: Arthropoda; class: Insecta; order: Hymenoptera; family: Megachilidae; genus: Megachile; subgenus: Callomegachile; specificEpithet: sculpturalis; scientificNameAuthorship: Smith, 1853; vernacularName: Giant Resin Bee; nomenclaturalCode: ICZN; taxonomicStatus: accepted; **Location:** continent: North America; country: United States; countryCode: US; stateProvince: Mississippi; county: Oktibbeha; locality: 3 mi East of Starkville; locationRemarks: Collected in Vitex agnus-castus flowers; verbatimCoordinates: 33°25’47”N, -88°44’01” W; verbatimLatitude: 33°25’47”N; verbatimLongitude: -88°44’01” W; **Event:** year: 2008; month: 7; day: 20; verbatimEventDate: Jul-20-2008; **Record Level:** type: PhysicalObject; institutionCode: MEM; basisOfRecord: PreservedSpecimen
- **Type status:** Other material. **Occurrence:** recordedBy: JoVonn G. Hill; individualCount: 2; sex: 1 male, 1 female; lifeStage: adult; occurrenceStatus: present; preparations: whole animal (pinned); disposition: in collection; **Taxon:** scientificName: *Megachile sculpturalis* Smith, 1853; kingdom: Animalia; phylum: Arthropoda; class: Insecta; order: Hymenoptera; family: Megachilidae; genus: Megachile; subgenus: Callomegachile; specificEpithet: sculpturalis; scientificNameAuthorship: Smith, 1853; vernacularName: Giant Resin Bee; nomenclaturalCode: ICZN; taxonomicStatus: accepted; **Location:** continent: North America; country: United States; countryCode: US; stateProvince: Mississippi; county: Oktibbeha; locality: 3 mi East of Starkville; locationRemarks: Collected in Vitex agnus-castus flowers; verbatimCoordinates: 33°25’47”N, -88°44’01” W; verbatimLatitude: 33°25’47”N; verbatimLongitude: -88°44’01” W; **Event:** year: 2008; month: 7; day: 27; verbatimEventDate: Jul-27-2008; **Record Level:** type: PhysicalObject; institutionCode: MEM; basisOfRecord: PreservedSpecimen
- **Type status:** Other material. **Occurrence:** recordedBy: JoVonn G. Hill; individualCount: 5; sex: male; lifeStage: adult; occurrenceStatus: present; preparations: whole animal (pinned); disposition: in collection; **Taxon:** scientificName: *Megachile sculpturalis* Smith, 1853; kingdom: Animalia; phylum: Arthropoda; class: Insecta; order: Hymenoptera; family: Megachilidae; genus: Megachile; subgenus: Callomegachile; specificEpithet: sculpturalis; scientificNameAuthorship: Smith, 1853; vernacularName: Giant Resin Bee; nomenclaturalCode: ICZN; taxonomicStatus: accepted; **Location:** continent: North America; country: United States; countryCode: US; stateProvince: Mississippi; county: Oktibbeha; locality: 3 mi East of Starkville; locationRemarks: Collected in Vitex agnus-castus flowers; verbatimCoordinates: 33°25’47”N, -88°44’01” W; verbatimLatitude: 33°25’47”N; verbatimLongitude: -88°44’01” W; **Event:** year: 2008; month: 7; day: 29; verbatimEventDate: Jul-29-2008; **Record Level:** type: PhysicalObject; institutionCode: MEM; basisOfRecord: PreservedSpecimen
- **Type status:** Other material. **Occurrence:** recordedBy: Christopher T. Werle; individualCount: 3; sex: male; lifeStage: adult; occurrenceStatus: present; preparations: whole animal (pinned); disposition: in collection; **Taxon:** scientificName: *Megachile sculpturalis* Smith, 1853; kingdom: Animalia; phylum: Arthropoda; class: Insecta; order: Hymenoptera; family: Megachilidae; genus: Megachile; subgenus: Callomegachile; specificEpithet: sculpturalis; scientificNameAuthorship: Smith, 1853; vernacularName: Giant Resin Bee; nomenclaturalCode: ICZN; taxonomicStatus: accepted; **Location:** continent: North America; country: United States; countryCode: US; stateProvince: Mississippi; county: Pear River; municipality: McNeill; locationRemarks: emerged from wooden trap nests, 3/8” holes; **Identification:** identifiedBy: Blair J. Sampson; **Event:** samplingProtocol: wooden trap nests, 3/8” holes; year: 2011; month: 7; day: 2-7; verbatimEventDate: VI-2-7-2011; **Record Level:** type: PhysicalObject; institutionCode: MEM; basisOfRecord: PreservedSpecimen
- **Type status:** Other material. **Occurrence:** recordedBy: Christopher T. Werle; individualCount: 4; sex: male; lifeStage: adult; occurrenceStatus: present; preparations: whole animal (pinned); disposition: in collection; **Taxon:** scientificName: *Megachile sculpturalis* Smith, 1853; kingdom: Animalia; phylum: Arthropoda; class: Insecta; order: Hymenoptera; family: Megachilidae; genus: Megachile; subgenus: Callomegachile; specificEpithet: sculpturalis; scientificNameAuthorship: Smith, 1853; vernacularName: Giant Resin Bee; nomenclaturalCode: ICZN; taxonomicStatus: accepted; **Location:** continent: North America; country: United States; countryCode: US; stateProvince: Mississippi; county: Pear River; municipality: McNeill; locationRemarks: emerged from wooden trap nests, 3/8” holes; **Identification:** identifiedBy: Blair J. Sampson; **Event:** samplingProtocol: wooden trap nests, 3/8” holes; year: 2011; month: 7; day: 2-7; verbatimEventDate: VI-2-7-2011; **Record Level:** type: PhysicalObject; institutionCode: BJS; basisOfRecord: PreservedSpecimen
- **Type status:** Other material. **Occurrence:** recordedBy: Ruth Tierce; individualCount: 2; sex: female; lifeStage: adult; occurrenceStatus: present; preparations: whole animal (pinned); disposition: in collection; **Taxon:** scientificName: *Megachile sculpturalis* Smith, 1853; kingdom: Animalia; phylum: Arthropoda; class: Insecta; order: Hymenoptera; family: Megachilidae; genus: Megachile; subgenus: Callomegachile; specificEpithet: sculpturalis; scientificNameAuthorship: Smith, 1853; vernacularName: Giant Resin Bee; nomenclaturalCode: ICZN; taxonomicStatus: accepted; **Location:** continent: North America; country: United States; countryCode: US; stateProvince: Mississippi; county: Yalobusha; locality: 4 mi NE of Coffeeville; locationRemarks: on back door of house; **Event:** samplingProtocol: at large; year: 2004; month: 8; day: 6; verbatimEventDate: IIX-6-2004; **Record Level:** type: PhysicalObject; institutionCode: MEM; basisOfRecord: PreservedSpecimen
- **Type status:** Other material. **Occurrence:** recordedBy: C. Zirkle; individualCount: 1; sex: male; lifeStage: adult; occurrenceStatus: present; preparations: whole animal (pinned); disposition: in collection; **Taxon:** scientificName: *Megachile sculpturalis* Smith, 1853; kingdom: Animalia; phylum: Arthropoda; class: Insecta; order: Hymenoptera; family: Megachilidae; genus: Megachile; subgenus: Callomegachile; specificEpithet: sculpturalis; scientificNameAuthorship: Smith, 1853; vernacularName: Giant Resin Bee; nomenclaturalCode: ICZN; taxonomicStatus: accepted; **Location:** continent: North America; country: United States; countryCode: US; stateProvince: Missouri; county: Barry; locationRemarks: cleared ridge top for cattle grazing, Flowering plants nearby: water hemlock, poke, mullein, blackberries, and Ozark chinquapin; verbatimCoordinates: N36°37'54.770" W93°49'05.382"; verbatimLatitude: N36°37'54.770"; verbatimLongitude: W93°49'05.382"; decimalLatitude: 36.631881; decimalLongitude: -93.818162; **Event:** samplingProtocol: blue vane trap; year: 2015; verbatimEventDate: June 28- July 3, 2015; **Record Level:** type: PhysicalObject; institutionCode: UAAM; basisOfRecord: PreservedSpecimen
- **Type status:** Other material. **Occurrence:** recordedBy: C. Zirkle; individualCount: 2; sex: 1 male, 1 female; lifeStage: adult; occurrenceStatus: present; preparations: whole animal (pinned); disposition: in collection; **Taxon:** scientificName: *Megachile sculpturalis* Smith, 1853; kingdom: Animalia; phylum: Arthropoda; class: Insecta; order: Hymenoptera; family: Megachilidae; genus: Megachile; subgenus: Callomegachile; specificEpithet: sculpturalis; scientificNameAuthorship: Smith, 1853; vernacularName: Giant Resin Bee; nomenclaturalCode: ICZN; taxonomicStatus: accepted; **Location:** continent: North America; country: United States; countryCode: US; stateProvince: Missouri; county: Barry; locationRemarks: cleared ridge top for cattle grazing, Flowering plants nearby: water hemlock and Ozark chinquapin; verbatimCoordinates: N36°37'38.993" W93°49'11.257"; verbatimLatitude: N36°37'38.993"; verbatimLongitude: W93°49'11.257"; decimalLatitude: 36.627498; decimalLongitude: -93.819794; **Event:** samplingProtocol: blue vane trap; year: 2015; month: 6; verbatimEventDate: June 21 - 28, 2015; **Record Level:** type: PhysicalObject; institutionCode: UAAM; basisOfRecord: PreservedSpecimen
- **Type status:** Other material. **Occurrence:** recordedBy: Bruce Schutte; individualCount: 1; sex: female; lifeStage: adult; occurrenceStatus: present; preparations: whole animal (pinned); disposition: in collection; **Taxon:** scientificName: *Megachile sculpturalis* Smith, 1853; kingdom: Animalia; phylum: Arthropoda; class: Insecta; order: Hymenoptera; family: Megachilidae; genus: Megachile; subgenus: Callomegachile; specificEpithet: sculpturalis; scientificNameAuthorship: Smith, 1853; vernacularName: Giant Resin Bee; nomenclaturalCode: ICZN; taxonomicStatus: accepted; **Location:** continent: North America; country: United States; countryCode: US; stateProvince: Missouri; county: Lincoln; locality: Cuivre River State Park; **Identification:** identifiedBy: Mike Arduser; **Event:** year: 2006; month: 6; verbatimEventDate: Jun-2006; **Record Level:** type: PhysicalObject; institutionCode: MA; basisOfRecord: PreservedSpecimen
- **Type status:** Other material. **Occurrence:** recordedBy: Mike Arduser; individualCount: 1; sex: male; lifeStage: adult; occurrenceStatus: present; preparations: whole animal (pinned); disposition: in collection; **Taxon:** scientificName: *Megachile sculpturalis* Smith, 1853; kingdom: Animalia; phylum: Arthropoda; class: Insecta; order: Hymenoptera; family: Megachilidae; genus: Megachile; subgenus: Callomegachile; specificEpithet: sculpturalis; scientificNameAuthorship: Smith, 1853; vernacularName: Giant Resin Bee; nomenclaturalCode: ICZN; taxonomicStatus: accepted; **Location:** continent: North America; country: United States; countryCode: US; stateProvince: Missouri; county: Pettis; municipality: Drover's Prairie; locality: South of Sedalia about 10 miles; **Identification:** identifiedBy: Mike Arduser; **Event:** year: 2010; month: 7; day: 10; verbatimEventDate: Jul-10-2010; **Record Level:** type: PhysicalObject; institutionCode: MA; basisOfRecord: PreservedSpecimen
- **Type status:** Other material. **Occurrence:** recordedBy: George Diehl Jr. & Mike Arduser; individualCount: 1; sex: female; lifeStage: adult; occurrenceStatus: present; preparations: whole animal (pinned); disposition: in collection; **Taxon:** scientificName: *Megachile sculpturalis* Smith, 1853; kingdom: Animalia; phylum: Arthropoda; class: Insecta; order: Hymenoptera; family: Megachilidae; genus: Megachile; subgenus: Callomegachile; specificEpithet: sculpturalis; scientificNameAuthorship: Smith, 1853; vernacularName: Giant Resin Bee; nomenclaturalCode: ICZN; taxonomicStatus: accepted; **Location:** continent: North America; country: United States; countryCode: US; stateProvince: Missouri; county: St. Louis; municipality: St. Louis City; locality: City Museum; locationRemarks: emerged from old log house; **Identification:** identifiedBy: Mike Arduser; **Event:** year: 2004; month: 7; day: 5; verbatimEventDate: Jul-05-2004; **Record Level:** type: PhysicalObject; institutionCode: MA; basisOfRecord: PreservedSpecimen
- **Type status:** Other material. **Occurrence:** recordedBy: Jane Stevens; individualCount: 1; sex: female; lifeStage: adult; occurrenceStatus: present; preparations: whole animal (pinned); disposition: in collection; **Taxon:** scientificName: *Megachile sculpturalis* Smith, 1853; kingdom: Animalia; phylum: Arthropoda; class: Insecta; order: Hymenoptera; family: Megachilidae; genus: Megachile; subgenus: Callomegachile; specificEpithet: sculpturalis; scientificNameAuthorship: Smith, 1853; vernacularName: Giant Resin Bee; nomenclaturalCode: ICZN; taxonomicStatus: accepted; **Location:** continent: North America; country: United States; countryCode: US; stateProvince: Missouri; county: St. Louis; municipality: St. Louis City; locality: St. Louis Zoo; **Identification:** identifiedBy: Mike Arduser; **Event:** year: 2007; month: 6; verbatimEventDate: Jun-2007; **Record Level:** type: PhysicalObject; institutionCode: MA; basisOfRecord: PreservedSpecimen
- **Type status:** Other material. **Occurrence:** recordedBy: Zach Scott; individualCount: 1; sex: male; lifeStage: adult; occurrenceStatus: present; preparations: whole animal (pinned); disposition: in collection; **Taxon:** scientificName: *Megachile sculpturalis* Smith, 1853; kingdom: Animalia; phylum: Arthropoda; class: Insecta; order: Hymenoptera; family: Megachilidae; genus: Megachile; subgenus: Callomegachile; specificEpithet: sculpturalis; scientificNameAuthorship: Smith, 1853; vernacularName: Giant Resin Bee; nomenclaturalCode: ICZN; taxonomicStatus: accepted; **Location:** continent: North America; country: United States; countryCode: US; stateProvince: Rhode Island; county: Washington; municipality: Kingston; locality: University of Rhode Island East Farm; decimalLatitude: 31.620610; decimalLongitude: -94.647550; **Identification:** identifiedBy: Zach Scott; **Event:** year: 2014; month: 7; day: 30; verbatimEventDate: Jul-30-2014; **Record Level:** type: PhysicalObject; institutionCode: ZS; basisOfRecord: PreservedSpecimen
- **Type status:** Other material. **Occurrence:** recordedBy: A. Hays; individualCount: 1; sex: female; lifeStage: adult; occurrenceStatus: present; preparations: whole animal (pinned); disposition: in collection; **Taxon:** scientificName: *Megachile sculpturalis* Smith, 1853; kingdom: Animalia; phylum: Arthropoda; class: Insecta; order: Hymenoptera; family: Megachilidae; genus: Megachile; subgenus: Callomegachile; specificEpithet: sculpturalis; scientificNameAuthorship: Smith, 1853; vernacularName: Giant Resin Bee; nomenclaturalCode: ICZN; taxonomicStatus: accepted; **Location:** continent: North America; country: United States; countryCode: US; stateProvince: Tennessee; county: Henderson; municipality: Lexington; locationRemarks: taken in back yard; decimalLatitude: 35.681934; decimalLongitude: -88.365206; **Event:** samplingProtocol: By Hand, In Yard; year: 2015; month: 6; day: 22; verbatimEventDate: Jun-22-2015; **Record Level:** type: PhysicalObject; institutionCode: BBSL; basisOfRecord: PreservedSpecimen; informationWithheld: Street Address
- **Type status:** Other material. **Occurrence:** recordedBy: Beverly A. Smith; individualCount: 1; sex: female; lifeStage: adult; occurrenceStatus: present; preparations: whole animal (pinned); disposition: in collection; **Taxon:** scientificName: *Megachile sculpturalis* Smith, 1853; kingdom: Animalia; phylum: Arthropoda; class: Insecta; order: Hymenoptera; family: Megachilidae; genus: Megachile; subgenus: Callomegachile; specificEpithet: sculpturalis; scientificNameAuthorship: Smith, 1853; vernacularName: Giant Resin Bee; nomenclaturalCode: ICZN; taxonomicStatus: accepted; **Location:** continent: North America; country: United States; countryCode: US; stateProvince: Tennessee; county: Rutherford; locality: Stone's River Glade; locationRemarks: Collected on Rudbeckia sp. in cedar glade; verbatimCoordinates: 35°52'24" N 86°26'09" W; verbatimLatitude: 35°52'24" N; verbatimLongitude: 86°26'09" W; decimalLatitude: 35.873333; decimalLongitude: -86.435833; **Event:** year: 2009; month: 7; day: 23; verbatimEventDate: VII-23-2009; **Record Level:** type: PhysicalObject; institutionCode: MEM; basisOfRecord: PreservedSpecimen
- **Type status:** Other material. **Occurrence:** recordedBy: Dan Bennett; individualCount: 2; sex: 1 male, 1 female; lifeStage: adult; occurrenceStatus: present; preparations: whole animal (pinned); disposition: in collection; **Taxon:** scientificName: *Megachile sculpturalis* Smith, 1853; kingdom: Animalia; phylum: Arthropoda; class: Insecta; order: Hymenoptera; family: Megachilidae; genus: Megachile; subgenus: Callomegachile; specificEpithet: sculpturalis; scientificNameAuthorship: Smith, 1853; vernacularName: Giant Resin Bee; nomenclaturalCode: ICZN; taxonomicStatus: accepted; **Location:** continent: North America; country: United States; countryCode: US; stateProvince: Texas; county: Nacogdoches; locality: Steven F. Austin State University Campus; verbatimElevation: 97 m; locationRemarks: on Vitex agnus-castus flowers (chaste tree); verbatimCoordinates: 31.62061°; - 94.64755°; verbatimLatitude: 31.62061°; verbatimLongitude: -94.64755°; decimalLatitude: 31.620610; decimalLongitude: -94.647550; **Identification:** identifiedBy: Dan Bennett; **Record Level:** type: PhysicalObject; institutionCode: SFAC; basisOfRecord: PreservedSpecimen
- **Type status:** Other material. **Occurrence:** recordedBy: Richard Brown; individualCount: 1; lifeStage: adult; occurrenceStatus: present; **Taxon:** scientificName: *Megachile sculpturalis* Smith, 1853; kingdom: Animalia; phylum: Arthropoda; class: Insecta; order: Hymenoptera; family: Megachilidae; genus: Megachile; subgenus: Callomegachile; specificEpithet: sculpturalis; scientificNameAuthorship: Smith, 1853; vernacularName: Giant Resin Bee; nomenclaturalCode: ICZN; taxonomicStatus: accepted; **Location:** continent: North America; country: United States; countryCode: US; stateProvince: Mississippi; county: Noxubee; locality: Noxubee National Wildlife Refuge; **Record Level:** type: Event; basisOfRecord: HumanObservation
- **Type status:** Other material. **Occurrence:** recordedBy: John Pascarella; individualCount: 1; lifeStage: adult; occurrenceStatus: present; **Taxon:** scientificName: *Megachile sculpturalis* Smith, 1853; kingdom: Animalia; phylum: Arthropoda; class: Insecta; order: Hymenoptera; family: Megachilidae; genus: Megachile; subgenus: Callomegachile; specificEpithet: sculpturalis; scientificNameAuthorship: Smith, 1853; vernacularName: Giant Resin Bee; nomenclaturalCode: ICZN; taxonomicStatus: accepted; **Location:** continent: North America; country: United States; countryCode: US; stateProvince: Texas; county: Huntsville; locality: Sam Houston State University; **Event:** year: 2014; month: 7; **Record Level:** type: Event; basisOfRecord: HumanObservation
- **Type status:** Other material. **Occurrence:** recordedBy: Jeff Harris; individualCount: 1; lifeStage: adult; occurrenceStatus: present; **Taxon:** scientificName: *Megachile sculpturalis* Smith, 1853; kingdom: Animalia; phylum: Arthropoda; class: Insecta; order: Hymenoptera; family: Megachilidae; genus: Megachile; subgenus: Callomegachile; specificEpithet: sculpturalis; scientificNameAuthorship: Smith, 1853; vernacularName: Giant Resin Bee; nomenclaturalCode: ICZN; taxonomicStatus: accepted; **Location:** continent: North America; country: United States; countryCode: US; stateProvince: Mississippi; county: Neshoba; municipality: Philadelphia; **Event:** year: 2015; month: 7; **Record Level:** type: Event; basisOfRecord: HumanObservation

#### Distribution

**Figure 2.**
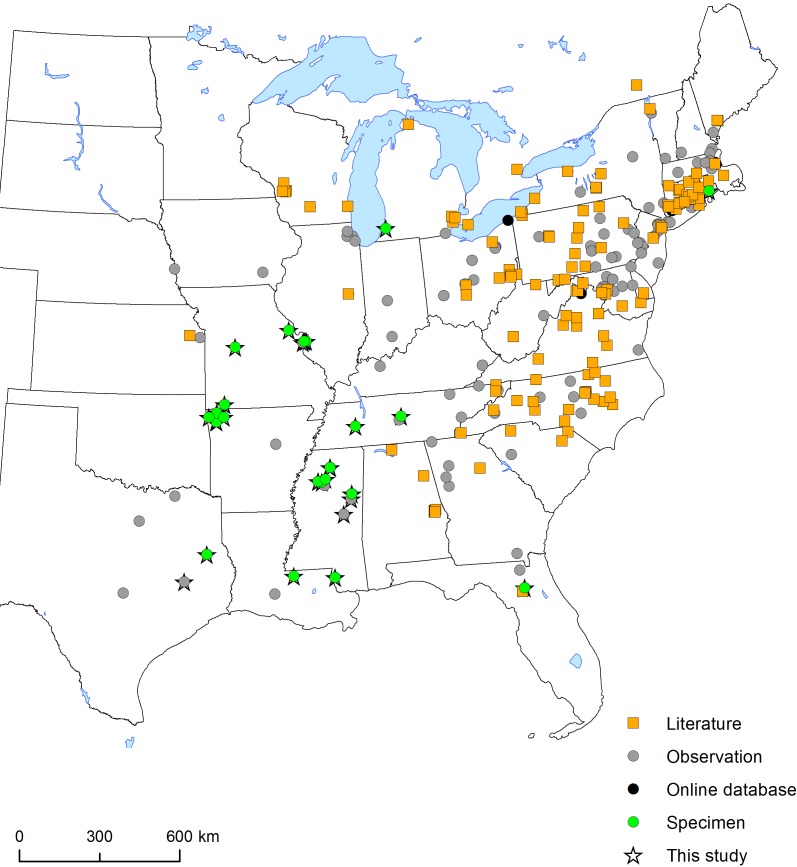
Map of eastern US showing newly reported records collected from new (starred) specimen data presented here (green circles, observation records from personal communications, Bugguide and GBIF iNaturalist records (grey circles), the GBIF online specimen database (black circles) and in the literature (orange squares). See Suppl. material 1.

**Figure 3.**
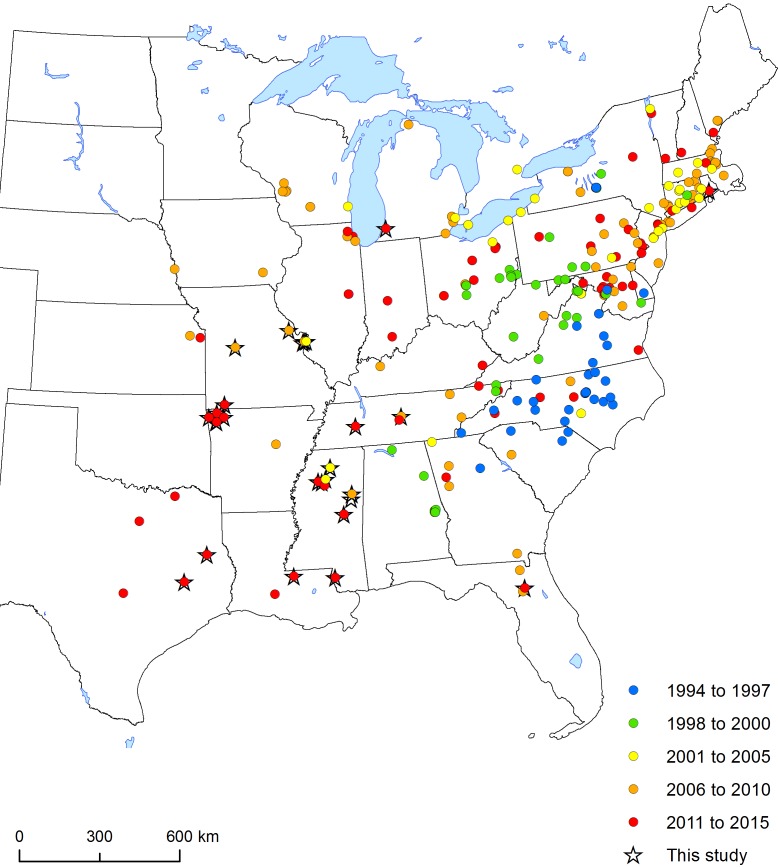
Map of eastern US showing collection/observation dates for *M. sculpturalis*. Data compiled from new reports in this study (starred), as well as other sources. See Suppl. material 1.

Specimen and observational data presented here expand the known distribution of *M. sculpturalis* to every state east of the Mississippi River and several western states including Arkansas, Iowa, Kansas, Louisiana, Missouri, Nebraska, and Texas (Fig. 2). Hinojosa-Díaz et al. (2005) used niche modeling to predict the expansion of the range in the United States, and current data continue to support that model (e.g., Hinojosa-Diaz 2008). While specimen records had previously been reported from Tennessee in the literature (Mangum and Sumner 2003), the records presented were in the far eastern portion of the state. Museum records now extend the distribution west within the state and include *Rudbeckia* sp. (Asteraceae/Compositae) as a new floral host from specimen data presented here. Record of *M. sculpturalis* from Rhode Island was anecdotally mentioned in Maier (2005) but never confirmed with collected specimens. The first record of *M. sculpturalis* west of the Mississippi River dates to 2004 (♀, St. Louis, MO) and is reported here for the first time. An examination of collections from Oklahoma did not yield any records in that state, in spite of records nearby in Arkansas, Missouri, and Texas. Records presented here and in other manuscripts show clear geographic expansion over time (Fig. 3), but it appears that the formal documentation of records lags behind the actual expansion.

## Discussion

Introduced bees can have a variety of undesirable effects including competition with native bees for both nesting sites and floral resources, transmission of diseases to native species, changes in seed set of native plants, and pollination of introduced plants (Goulson 2003). Negative interactions have been observed between *M. sculpturalis* and the native *X. virginica*, though long-term effects of these aggressive behaviors on *Xylocopa* populations are unknown (Laport and Minckley 2012, Roulston and Malfi 2012). Nesting sites made by *X. virginica* were present at field locations where specimens were collected in both Paynes and Scobey, MS, and both species were observed simultaneously during the summer of 2015. Specimens of *M. sculpturalis* collected from Pearl River Co., MS emerged from 3 of 17 (18%) occupied wooden trap nests constructed of 3/8” holes while trying to collect *Osmia* sp. It is highly likely that negative interactions exist with not only *Xylocopa*, but with *Osmia* and other cavity nesting species that would utilize a nest chamber of a similar diameter.

Other non-native bee populations preferentially pollinate floral resources that have also been introduced (Hanley and Goulson 2003, Morales and Aizen 2002). Host plant records for *M. sculpturalis* indicate that the majority of published records have been collected from plants not native to North America (Table 1). Current floral host plant associations include 43 species (30 species and an additional 13 genera without species names) in 21 families. While pollination is an important ecosystem service and provides an economic benefit to agricultural production, none of the plants listed in Table 1 are considered to be prominent crops in the mid-or gulf-south.

Specimens of *M. sculpturalis* were intercepted at the port of Baltimore in cargo shipped from Japan previous to establishment in 1968 and 1976 (Batra 1998). As cavity nesters that actively utilize holes made by other species, range expansion within the United States likely includes movement in wood. Distribution on wood in various forms is one of the most common methods by which invasive species are spread (Moore 2005). Additional locations with established populations of *X. virginica* were identified in Washington, Bolivar, and Sunflower Counties, MS and Chicot Co., AR but no *M. sculpturalis* were observed, suggesting that distributions are not continuous. The current known distribution of *M. sculpturalis* appears to be limited to locations where someone has noticed that these flying insects are not the commonly encountered *Xylocopa*. At several collection locations in MS, property owners were unaware of the presence of *M. sculpturalis* and allowed us to examine *Xylocopa* nesting locations, revealing new distributional points. This suggests that especially in locations where multiple species occur, they are easily mistaken for *Xylocopa*. Of the records examined from the mid- and gulf-south, it appears that specimens are rarely collected by non-specialists.

Large distinctive bees that have been introduced to an area, like *M. sculpturalis* and others, can be monitored through online entomology and photography groups (e.g. Bugguide 2015) often before peer reviewed literature can be published. Eight of the new host plant records presented (Table 1) are from photographs posted on Bugguide, while traditional collection information specimen data provided only one new floral record.

## Supplementary Material

Supplementary material 1 Supplementary table 1 - occurence data Data type: occurences
Brief description: spreadsheet containing occurence information for *M. sculpturalis* specimens.
File: oo_57781.xlsx K. A. Parys, A. D. Tripodi, B. J. Sampson

XML Treatment for Megachile (Callomegachile) sculpturalis

## Figures and Tables

**Figure 1a.**
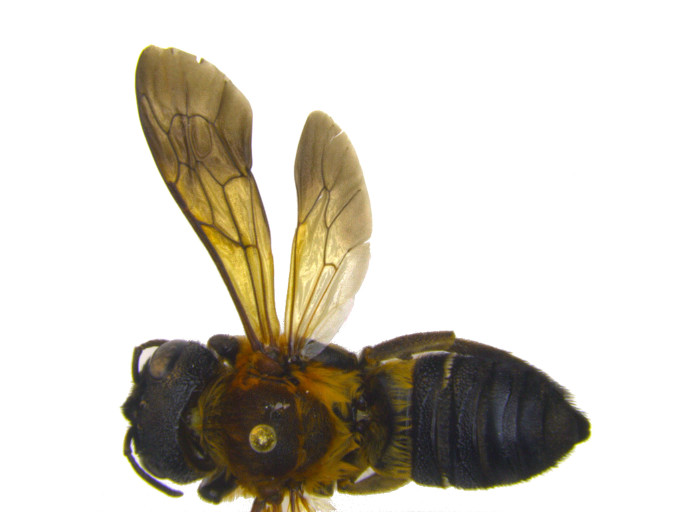
dorsal view female.

**Figure 1b.**
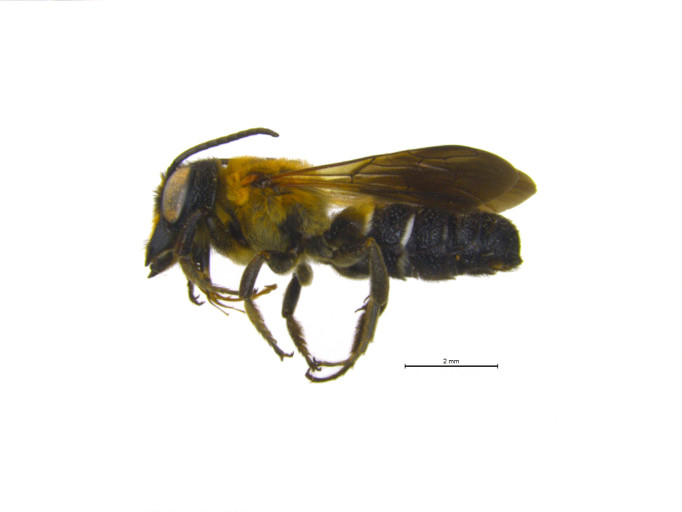
lateral view male. Scale bar = 2mm.

**Figure 1c.**
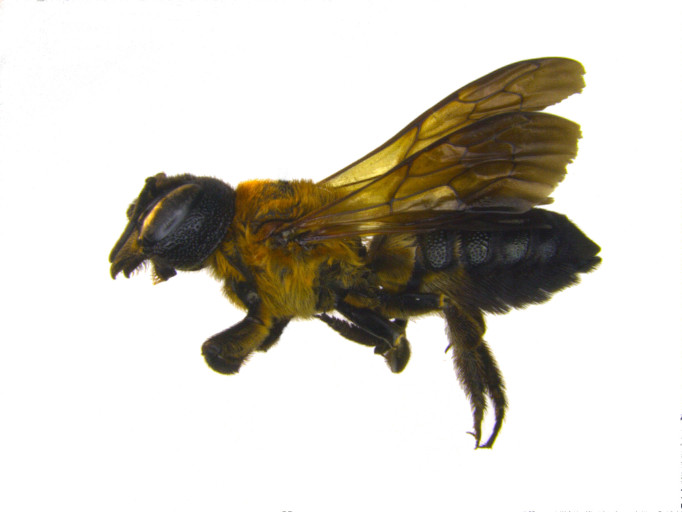
lateral view female.

**Table 1.** List of host plants associated with *M. sculpturalis*. Records are taken from scientific literature, photographs on Bugguide that included plant associations, and specimen data. Information concerning whether plants are native to North America and standardized plant names were found using The Plant List (2015) and references therein

**Host Plant**	**Status in North America**	**Data Location**
**Apiaceae**		
*Daucus carota* L.	Introduced	Bugguide (e.g. Wilder (2009))
**Apocyanaceae**		
*Asclepias* sp.	--	Mangum and Sumner (2003)
*Asclepias syriaca* L.	Native	Ascher (2001), Maier (2005)
**Asteraceae/Compositae**		
*Cirsium vulgare* (Savi) Ten.	Introduced	Ascher (2001)
*Liatris* sp.	--	Bugguide (e.g. Phillips (2014))
*Liatris spicata* (L.) Willd.	Native	Bugguide (e.g. Balaban and Balaban (2011))
*Rudbeckia* sp.	--	In specimen data above (MEM)
*Solidago* sp.	--	Maier (2005)
**Bigonaniaceae**		
*Catalpa* sp.	--	Norden (2008)
*Catalpa speciosa* (Warder) Engelm.	Native	Batra (1998), Mangum and Sumner (2003)
**Boraginaceae**		
*Echium vulgare* L.	Introduced	Bugguide (e.g. Harrison (2014))
**Crassulaceae**		
*Sedum* sp.	--	Bugguide (e.g. Moisset (2007))
**Ericaceae**		
*Oxydendrum arboreum* (L.) DC.	Native	Batra (1998), Mangum and Sumner (2003)
**Fagaceae**		
*Castanea* sp.	--	Quaranta et al. (2014)
**Lamiaceae**		
*Lavandula* sp.	--	Vereecken and Barbier (2009)
*Origanum laevigatum* Boiss.	Introduced	Bugguide (e.g. Roos (2013))
*Perovskia artemisioides* Boiss	Introduced	Ascher (2001)
*Perovskia atriplicifolia* Benth.	Introduced	Mazurkiewicz (2010)
*Pycnanthemum* sp.	--	Bugguide (e.g. Simpson and Simpson (2012))
*Vitex* sp.	--	Mangum and Sumner (2003)
*Vitex agnus-castus* L.	Introduced	Hall and Ascher (2010), In specimen data above (MEM)
**Leguminosea**		
*Dunbaria villosa* (Thunb.) Makino = *Dumbaria villosa*	Introduced	Batra 1998, Iwata 1933
*Lathyrus latifolius* L.	Introduced	Ascher (2001), Mangum and Sumner (2003), O'Brien and Craves (2008), Hinojosa-Díaz et al. (2005)
*Lespedza* sp.	--	Batra (1998)
*Melilotus albus* Medik.	Introduced	Ascher (2001), Paiero and Buck (2003)
*Millettia japonica* (Siebold &Zucc.) A. Gray	Introduced	Batra (1998), Iwata (1933)
*Phaseolus vulgaris* L.	Introduced	Batra (1998), Iwata (1933)
*Pueraria lobata* (Willd.) Ohwi.	Introduced	Mangum and Brooks (1997)
Pueraria montana var. lobata (Willd.) Sanjappa & Pradeep	Introduced	Iwata (1933)
*Robinia* sp.	--	Quaranta et al. (2014)
*Securigera varia* (L.) Lassen = *Coronilla varia* L.	Introduced	Ascher (2001)
*Styphnolobium japonicum* (L.) Schott = *Sophora japonica* L.	Introduced	Hinojosa-Díaz et al. (2005), Mangum and Sumner (2003), Matteson et al. (2008)
*Vigna unguiculata* (L.) Walp = Vigna catiang var. sinensis = *Vigna sinensis* (L.) Savi ex Hassk.	Introduced	Batra (1998), Iwata (1933)
**Lythraceae**		
*Lagerstroemia indica* L.	Introduced	Batra (1998)
*Lythrum salicaria* L.	Introduced	Mangum and Sumner (2003), Maier (2005), Maier (2009), O'Brien and Craves (2008)
**Myrtaceae**		
*Eucalyptus* sp.	--	Quaranta et al. (2014)
**Oleaceae**		
*Chionanthus* sp.	--	Norden (2008)
*Ligustrum* sp.	--	Norden (2008), Quaranta et al. (2014)
*Ligustrum lucidum* W.T. Aiton	Introduced	Mangum and Sumner (2003), Hinojosa-Díaz et al. (2005)
*Ligustrum vulgare* L.	Introduced	Batra (1998)
**Plantaginaceae**		
*Veronicastrum virginicum* (L.) Farw.	Native	Paiero and Buck (2003), O'Brien and Craves (2008)
**Plumbaginaceae**		
*Limonium carolinianum* (Walter) Britton	Native	Maier (2005)
**Rosaceae**		
*Rubus* sp.	--	Quaranta et al. (2014)
**Rubiaceae**		
*Cephalanthus* sp.	--	Bugguide (e.g. Borchelt (2015))
**Rutaceae**		
*Citrus japonica* Thunb. = *Fortunella margarita* Swingle	Introduced	Batra (1998)
**Sapindaceae**		
*Koelreuteria paniculata* Laxm.	Introduced	Mangum and Brooks (1997), Mangum and Sumner (2003), Hinojosa-Díaz et al. (2005), Batra (1998), In specimen data above (UMIC)
**Scrophulariaceae**		
*Buddleja* sp. = *Buddleia* sp.	--	Wolf and Ascher (2008)
*Buddleja davidii* Franch	Introduced	Mangum and Sumner (2003)
*Verbascum thapsus* L.	Introduced	Ascher (2001)
**Vitaceae**		
*Parthenocissus* sp.	--	Quaranta et al. (2014)

## References

[B1889205] Amiet F. (2012). Die Blattschneiderbiene *Megachile
sculpturalis* Smith, 1853 (Hymenoptera: Apidae) nun auch in der Schweiz. Entomo Helvetica.

[B1889327] Ascher JA (2001). *Hylaeus
hyalinatus* Smith, a European Bee New to North America, with Notes on Other Adventive Bees (Hymenoptera: Apoidea). Proceedings of the Entomological Society of Washington.

[B1889347] Ascher JS, Pickering J Discover Life bee species guide and world checklist (Hymenoptera: Apoidea: Anthophila). http://www.discoverlife.org/mp/20q?guide=Apoidea_species.

[B1889356] Balaban J, Balaban J Bee on Liatris spicata - Megachile sculpturalis. http://bugguide.net/node/view/597325/bgimage.

[B1889365] Batra S (1998). Biology of the giant resin bee, *Megachile
sculpturalis* Smith, a conspicuous new immigrant in Maryland. Maryland Naturalist.

[B1889375] Borchelt R Unidentified bee on Cephalanthus, 2015 Jun 20, Kent Co MD Eastern Neck NWR - Megachile sculpturalis. http://bugguide.net/node/view/1087619/bgimage.

[B1889384] Bugguide http://www.bugguide.net.

[B1889393] Cane James H, Strickler K, Cane J H (2003). Exotic Nonsocial Bees (Hymenoptera: Apiformes) in North America: Ecological Implications. For Nonnative Crops, "Whence Pollinators of the Future?".

[B1889407] Droge S http://www.pwrc.usgs.gov/nativebees/Handy%20Bee%20Manual/Handy%20Bee%20Manual.pdf. The Very Handy Bee Manual.

[B1889416] Evenhuis N Abbreviations for insect and spider collections of the world. http://hbs.bishopmuseum.org/codens/codens-inst.html.

[B1889425] Genaro J A (1996). Key to the genus *Megachile*, *Chalicodoma* group (Hymenoptera: Megachilidae) in Cuba. Revista de Biologia Tropical.

[B1889435] Gihr C, Westrich P (2013). Breeding record of *Megachile
sculpturalis* (giant resin bee) in Southern France (Hymenoptera, Apidae). Eucera.

[B1889445] Goulson D (2003). Effect of Introduced Bees on Native Ecosystems. Annual Review of Ecology Evolution and Systematics.

[B2034369] GRBio The Global Registry of Biodiversity Repositories. http://grbio.org/.

[B1889455] Hall H. Glenn, Ascher John S. (2010). Surveys of Bees (Hymenoptera: Apoidea: Anthophila) in Natural Areas of Alachua County in North-Central Florida. Florida Entomologist.

[B1889465] Hanley M E, Goulson D (2003). Introduced weeds pollinated by introduced bees: Cause or effect?. Weed Biology and Management.

[B1889475] Harrison E Unidentified bee/wasp/fly - Megachile sculpturalis. http://bugguide.net/node/view/1025233/bgimage.

[B1889317] Hinojosa-Díaz Ismael (2008). The giant resin bee making its way west: First record in Kansas (Hymenoptera: Megachilidae). ZooKeys.

[B1889498] Hinojosa-Díaz I A, Yáñez-Ordóñez O, Chen G, Peterson A T, Engel M S (2005). The North American Invasion of the Giant Resin Bee (Hymenoptera: Megachilidae). Journal of Hymenoptera Research.

[B1889509] Iwata K (1933). Studies on the nesting habitats and parasites of *Megachile
sculpturalis*. Mushi.

[B1889519] Kondo T, Williams M L, Minckley R (2000). Giant Resin Bees@ Exotic species makes it way from east coast to Alabama. Alabama Agricultural Experiment Station Highlights of Research.

[B1889529] Laport Robert G., Minckley Robert L. (2012). Occupation of Active *Xylocopa
virginica* Nests by the Recently Invasive *Megachile
sculpturalis* in Upstate New York. Journal of the Kansas Entomological Society.

[B1889549] Maier C (2005). First records of alien insects in Connecticut (Orthoptera: Tettigoniidae; Coleoptera: Buprestidae, Chrysomelidae; Diptera: Rhagionidae, Tephritidae; Hymenoptera: Megachilidae). Proceedings of the Entomological Society of Washington.

[B1889559] Maier C (2009). New Distributional Records of Three Alien Species of Megachilidae (Hymenoptera) from Connecticut and Nearby States. Proceedings of the Entomological Society of Washington.

[B1889569] Mangum W A, Brooks R W (1997). First records of Megachile (Callomegachile) sculpturalis Smith (Hymenoptera: Megachilidae) in the Continental United States. Journal of the Kansas Entomological Society.

[B1889579] Mangum W A, Sumner S (2003). A survey of the North American range of Megachile (Callomegachile) sculpturalis, an adventive species in North America. Journal of the Kansas Entomological Society.

[B1889589] Matteson Kevin C., Ascher John S., Langellotto Gail A. (2008). Bee Richness and Abundance in New York City Urban Gardens. Annals of the Entomological Society of America.

[B1889599] Mazurkiewicz M (2010). The Giant Resin Bee, *Megachile
sculpturalis*, in Maine: a New State Record. The Maine Entomologist.

[B1889609] Michener C D (2007). The Bees of the World.

[B1889628] Moisset B Giant resin bee - Megachile sculpturalis - Male. http://bugguide.net/node/view/161659/bgimage.

[B1889637] Moore B (2005). Alien Invasive Species: Impacts on Forests and Forestry.

[B1889646] Morales Carolina L., Aizen Marcelo A. (2002). Does Invasion of Exotic Plants Promote Invasion of Exotic Flower Visitors? A Case Study from the Temperate Forests of the Southern Andes. Biological Invasions.

[B1889656] Norden Beth B. (2008). A Checklist of the Bees (Insecta: Hymenoptera) and Their Floral Hosts at Plummers Island, Maryland. Bulletin of the Biological Society of Washington.

[B1889666] O'Brien M F, Craves J (2008). *Megachile
sculpturalis* Smith - A New Bee for Michigan (Hymenoptera: Megachile). Newsletter of the Michigan Entomological Society.

[B1889676] Paiero S M, Buck M (2003). The Giant Resin Bee, *Megachile
sculpturalis* Smith, and other newly introduced and newly recorded native Megachilidae and Andrenidae (Apoidea) from Ontario. Journal of the Entomological Society of Ontario.

[B1889686] Pasteels J J (1965). Revision des Megachilidae (Hymenoptera Apoidea) de l'Afrique noire. I. Les genres *Creightoniella*, *Chalicodoma* et *Megachile* (s. str.). Koninklijk Museum voor Midden-Afrika, Tervuren, België Annalen, Reeks In-8. Zoologische Wetenschappen.

[B1889696] Phillips P Resin bee? on liatris - Megachile sculpturalis. http://bugguide.net/node/view/981063/bgimage.

[B1889705] Quaranta M, Sommaruga A, Balzarini P, Felicioli A (2014). A new species for the bee fauna of Italy: *Megachile
sculpturalis* continues its colonization of Europe. Bulletin of Insectology.

[B1889715] Roos D Unidentified bee on ornamental oregano - Megachile sculpturalis.. http://bugguide.net/node/view/808917/bgimage.

[B1889539] Roulston T'ai, Malfi Rosemary (2012). Aggressive Eviction of the Eastern Carpenter Bee (*Xylocopa
virginica* (Linnaeus)) from its Nest by the Giant Resin Bee (*Megachile
sculpturalis* Smith). Journal of the Kansas Entomological Society.

[B1889724] Simberloff Daniel, Martin Jean-Louis, Genovesi Piero, Maris Virginie, Wardle David A., Aronson James, Courchamp Franck, Galil Bella, García-Berthou Emili, Pascal Michel, Pyšek Petr, Sousa Ronaldo, Tabacchi Eric, Vilà Montserrat (2013). Impacts of biological invasions: what's what and the way forward. Trends in Ecology & Evolution.

[B1889744] Simpson R, Simpson A Large Bee - Megachile sculpturalis. http://bugguide.net/node/view/618394/bgimage.

[B1889753] List The Plant A working list of all plant species (Version 1.1). http://www.theplantlist.org.

[B1889771] Tonietto R K, Ascher J S (2008). Occurance of the old world bee species *Hylaeus
hyalinatus*, *Anthidium
manicatum*, *Anthidium
oblongatum*, and *Megachile
sculpturalis*, and the native species *Coelioxys
banksi*, *Lasioglossum
michiganense*, and *Lasioglossum
zophops* in Illinois (Hymenoptera: Apoidea: Colletidae, Halictidae, Megachilidae). Great Lakes Entomologist.

[B1889781] Vereecken N J, Barbier E (2009). Premières données sur la présence de l’abeille asiatique Megachile (Callomegachile) sculpturalis Smith (Hymenoptera, Megachilidae) en Europe. Osmia.

[B1889791] Wilder C Giant Resin Bee - Megachile sculpturalis - Male. http://bugguide.net/node/view/311724/bgimage.

[B1889800] Wolf A T, Ascher J S (2008). Bees of Wisconsin (Hymenoptera: Apoidea: Anthophila). Great Lakes Entomologist.

